# Improved Distance Functions for Instance-Based Text Classification

**DOI:** 10.1155/2020/4717984

**Published:** 2020-11-22

**Authors:** Khalil El Hindi, Bayan Abu Shawar, Reem Aljulaidan, Hussien Alsalamn

**Affiliations:** ^1^Department of Computer Science, King Saud University, Riyadh, Saudi Arabia; ^2^Cypersecurity Department, School of Engineering, Al Ain University, Abu Dhabi, UAE

## Abstract

Text classification has many applications in text processing and information retrieval. Instance-based learning (IBL) is among the top-performing text classification methods. However, its effectiveness depends on the distance function it uses to determine similar documents. In this study, we evaluate some popular distance measures' performance and propose new ones that exploit word frequencies and the ordinal relationship between them. In particular, we propose new distance measures that are based on the value distance metric (VDM) and the inverted specific-class distance measure (ISCDM). The proposed measures are suitable for documents represented as vectors of word frequencies. We compare these measures' performance with their original counterparts and with powerful Naïve Bayesian-based text classification algorithms. We evaluate the proposed distance measures using the kNN algorithm on 18 benchmark text classification datasets. Our empirical results reveal that the distance metrics for nominal values render better classification results for text classification than the Euclidean distance measure for numeric values. Furthermore, our results indicate that ISCDM substantially outperforms VDM, but it is also more susceptible to make use of the ordinal nature of term-frequencies than VDM. Thus, we were able to propose more ISCDM-based distance measures for text classification than VDM-based measures. We also compare the proposed distance measures with Naïve Bayesian-based text classification, namely, multinomial Naïve Bayes (MNB), complement Naïve Bayes (CNB), and the one-versus-all-but-one (OVA) model. It turned out that when kNN uses some of the proposed measures, it outperforms NB-based text classifiers for most datasets.

## 1. Introduction

Text classification can be defined as the task of assigning a document to a category such as art, sport, and politics. The proliferation of online documents by the minute made automatic text classification an essential component of many online systems. Text classification has many real-world applications, including identifying relevant news articles, filtering spam e-mail messages [1], automatic indexing for information retrieval [2], e-mail routing [3], e-mail threading (sorting) [4], human-computer interaction [5], sentiment analysis, word sense disambiguation [6], and many other automated document processing applications. Machine learning algorithms can be used to train classifiers to classify documents automatically based on classified (labeled) instances. These methods are more effective than knowledge engineering methods, where human experts manually formulate rules for classifying documents [2].

Text classification is a challenging problem for machine learning algorithms [7, 8]; as a typical text classification dataset consists of thousands of features, many of which are redundant, and overfitting may occur. Many machine learning methods, however, perform well in text classification [2], including the Naïve Bayesian (NB) learning algorithms [9–11], instance-based learning [2, 8], and support vector machines SVM [12].

IBL is a simple and effective learning method in many application domains, including text classification [2, 8]. The kNN algorithm is a simple IBL algorithm. During training, it only stores all classified (labeled) instances. To classify a query instance, it retrieves the most similar *k* instance(s) and uses their classes and a voting mechanism to predict a class for the query instance (the class with the majority of votes). However, the performance of IBL depends on the similarity or distance measure used to determine the most similar (or nearest) instances [8]. Moreover, a distance function must be efficient to compute to be of practical use because every time we want to classify a new document, all training documents need to be ranked for similarity with it [2]. In this paper, we compare the value difference metric (VDM) [13, 14] and the inverted specific-class distance measure SCDM [15], which were designed to measure the distance between nominal attribute values. We also propose several improvements to these measures to enable them to make use of the extra information available when documents are represented as vectors of term-frequencies. This document representation method represents the frequency of each word that is used in a document and is essential for multinomial NB text classification [9]. Therefore, the proposed measures would not result in better performance for documents represented as binary features that only reflect whether terms occur or do not occur in a document. Moreover, the measures we present assume that each training document is labeled, which makes them unsuitable for unsupervised learning (clustering) methods. For distance measures that are suitable for unsupervised text classification, please see [16].

This paper is structured as follows. In Section 2 we discuss some related work.  Section 3 presents the proposed distance measures.  Section 4 compares the proposed measures with the original measures and with some Bayesian-based methods for text classification.

## 2. Related Work

This section discusses the distance measures and powerful Bayesian-based text classification methods that we use in our empirical comparisons.

### 2.1. Related Distance Measures

To determine the similarity between two instances, a distance measure is usually used. If the attributes are numeric or ordinal, the task is simple because the difference between two attribute values reflects how similar they are, and the Euclidian distance can be used as a measure of similarity between the two instances. The Euclidean distance metric is by far the most commonly used distance function [14] for ordinal attributes. It is defined as

(1) $$\begin{array}{c} E\left(x,y\right)=\sqrt{\sum_{a=1}^{m}{\left(x_{a}-y_{a}\right)}^{2}}. \end{array}$$

However, if the values are nominal (i.e., having no ordering relationship between them), the task is more complicated. The overlap metric (OM) is one of the most widely used distance metrics for nominal attributes [17]. It is based on the total Hamming distance for all nominal attributes. The Hamming distance between two nominal values is zero if the two values are equal and one otherwise. Thus, the OM distance between two instances *x* and *y* is simply a count of the number of attribute values for which the two instances differ.

On the other hand, the VDM metric considers two nominal values similar if they have similar classifications (i.e., they occur with the same classes). The VDM is thus more potent than the OM [14, 17]. For example, as illustrated in [14], when classifying some fruits and vegetables given their color attribute values, the colors red and green are more similar than red and blue. This is because many fruits and vegetables (i.e., classes) can be red or green.

In this work, we use the best-normalized version of the VDM as reported by Wilson and Martinez [14], which is defined as

(2) $$\begin{array}{c} d\left(x,y\right)=\sqrt{\sum_{a=1}^{m}\text{vdm}\left(x_{a},y_{a}\right)2} \end{array}$$

(3) $$\begin{array}{c} vdm\left(x_{a},y_{a}\right)=\sum_{c=1}^{C}\left(P\left(c/x_{a}\right)-P\left(c/y_{a}\right)\right), \end{array}$$

where

- *x* and *y* are two vectors (documents); typically, one is a training instance and the other is a vector that needs to be classified
- *x*_*a*_ and *y*_*a*_ are the values of attribute *a* in the vectors *x* and *y*, respectively
- *m* is the number of attributes
- and *C* is the number of classes (document categories)

Most recent attempts to enhance the VDM, such as the one dependence VDM (OVDM) [18], the augmented VDM (AVDM) [17], and the local VDM (LVDM) [19], focus on relaxing the attribute independence assumption that the VDM suffers from. The OVDM accomplishes this by building a Bayesian network to capture the dependence relationships between attributes, while the AVDM relaxes the dependence assumption by transforming the m-dimensional input vector into a *C* · *t*(*m*, 2)-dimensional space, where *C* is the number of classes, and *t*(*m*, 2) is the number of 2-combinations of a set of *m* elements. The AVDM then uses the Manhattan distance on the transformed space, as

(4) $$\begin{array}{c} \text{avdm}\left(x,y\right)=\sum_{i=1}^{m-1}\sum_{a=i+1}^{m}\overset{C}{\underset{c=1}{\sum }}\left|P\left(c/x_{a},x_{i}\right)-P\left(c/y_{a},y_{i}\right)\right|. \end{array}$$

As the AVDM is applied to a larger space, it increases the computational cost of the VDM [17], which may hinder its use in text classification, where documents are typically represented using a large number of features.

The LVDM relaxes the conditional independence assumption by estimating the conditional probabilities from the neighborhood of a query instance only. The intuition is that the conditional independence assumption is more likely to be valid in the neighborhood of a query instance than in the entire dataset. A decision tree produced using a distance-based attribute measure is used to determine the neighborhood of a query instance. This method, too, increases the computational cost because the probability estimations can only be done at a classification time when a query instance is available.

Other distance functions for nominal attributes include the SFM [20, 21] and MRM [22]. The SFM is defined as

(5) $$\begin{array}{c} \text{SFM}\left(x,y\right)=\sum_{c=1}^{C}\left(P\left(c/x\right)-P\left(c/y\right)\right), \end{array}$$

where *P*(*c*/*x*) and *P*(*c*/*y*) are usually estimated using NB. The MRM, which minimizes the risk of misclassification, is defined as

(6) $$\begin{array}{c} \text{MRM}\left(x,y\right)=\sum_{c=1}^{C}P\left(c/x\right)\left(1-P\left(c/y\right)\right). \end{array}$$

It is worth noting that using the SFM and MRM increases the classification time because they need to be computed when a query instance is available; besides, they use NB classifier *C* times for each training instance. On the other hand, the VDM for every pair of values can be computed during training, and thus it does not incur any additional classification time.

In [15], several distance functions for nominal values were proposed. Unlike the VDM, which fails to make use of the class of the training instance with which a query instance is being compared, these functions exploit the class of the training instance. They are called specific-class distance measures (SCDM). The intuition is that it may be misleading to consider two nominal values similar only because they occur with the same set of classes. For example, as illustrated in [15], many fruits and vegetables can have a sweet or acidic taste. Therefore, the VDM would consider the two nominal values sweet and sour similar, but this could be misleading when comparing a query instance with a sweet taste with a training instance of class lemon with a sour taste. As lemon can only be sour, the query instance cannot be a lemon, and therefore the distance between the two values should be as considerable as possible. However, the VDM would return a small distance, indicating that the two instances are similar, while they are not and the distance should be as large as possible. Among the different SCDM-based measures [15], the inverted SCDM (ISCDM) is probably the most powerful [15]. It is defined as

(7) $$\begin{array}{c} d\left(x,y\right)=\sqrt{\sum_{a=1}^{m}\text{ISCDM}{\left(x_{a},y_{a}\right)}^{2}}, \end{array}$$

(8) $$\begin{array}{c} \text{ISCDM}\left(x_{a},y_{a}\right)=\left\{\begin{array}{cc} 1,\text{if }x_{a}\text{ is missing,}\\ \text{0,}\text{if }x_{a}=y_{a},\\ 1-p\left(x_{a}|/|y_{\text{class}}\right), & \text{otherwise,} \end{array}\right. \end{array}$$

where *x* and *y* are the query and training instances, respectively. *y*_class_ is the class value of the training instance. The empirical studies performed in [15, 23] show that the ISCDM outperforms the VDM for many benchmark datasets. Moreover, as the ISCDM does not depend on the attribute value of the training instance, *y*_*a*_, it is less sensitive to missing values in the training set and more robust to nonclass attribute noise [15, 23].

### 2.2. Naïve Bayesian-Based Text Classification

Several Naïve Bayesian-based text classification algorithms were proposed in the literature, including multinomial Naïve Bayes (MNB) [9], complement Naïve Bayes (CNB) [24], and the one-versus-all-but-one (OVA) model [24]. MNB, CNB, and OVA are among the top-performing Bayesian-based methods that make use of the frequency of words in documents. They approach the accuracy of support vector machines and at the same time faster and easier to implement [24]. In this study, we compare some of the proposed distance measures with these methods.

MNB uses the following equation to classify a document:

(9) $$\begin{array}{c} C_{MNB}={\text{argmax}}_{c\in C}\left[\log p\left(c\right)+\sum_{i=1}^{m}f_{i}\log\left(p\left(w_{i}|/|c\right)\right)\right], \end{array}$$

where *C* is a set of all class values.

CNB uses the following equation to classify a document:

(10) $$\begin{array}{c} C_{cnb}={\text{argmax}}_{c\in C}\left[-\log p\left(\bar{c}\right)-\sum_{i=1}^{m}f_{i}\log\left(p\left(w_{i}|/|\bar{c}\right)\right)\right], \end{array}$$

where $\bar{c}$ refers to all classes except class *c*.

OVA uses the following equation to classify a document:

(11) $$\begin{array}{c} C_{ova}={\text{argmax}}_{c\in C}\left[\log p\left(c\right)-\log p\left(\overset{¯}{c}\right)-\overset{m}{\underset{i=1}{\sum }}f_{i}\left(\log p\left(w_{i}/c\right)-\log p\left(w_{i}|/|\bar{c}\right)\right)\right]. \end{array}$$

## 3. New VDM-Based and ISCDM-Based Distance Measures for Text Classification

In Section 2, we saw that most attempts to improve the VDM increase the classification time, which hinders their use for text classification, where we have a large number of documents, each with a large number of features (words). In this section, we modify the VDM and ISCDM to allow them to use the frequencies of terms (words) without increasing the classification time.

We believe that the distance functions can be improved if we modify them in a way that allows them to exploit the word-frequency representation of documents. In this representation, each document is represented as a vector of integer values; each represents the frequency of a word in the document. This representation is mainly used for the MNB [9], CNB, and OVA. In contrast, a binary (or Boolean) representation of documents is not concerned with how many times a word occurs in a document, but whether a word occurs in the document or not. Thus, the distance measures we propose are not suitable for a binary representation of documents.

### 3.1. Incorporating a Euclidean Element

Since documents are represented as vectors of integers representing word frequencies, the natural choice would be the Euclidean distance measure. However, our empirical experiments revealed that VDM and ISCDM, designed for nominal values, achieve far more superior performance than the Euclidean distance measure for text classification. We used Euclidean distance ((1)) as a measure of similarity for the kNN algorithm (*k* = 3) and evaluated it using 18 benchmark text classification datasets (described in Table 1), but it gave a poor overall average classification accuracy of 63.74%. This is much lower than the average classification accuracies we obtained using the VDM and ISCDM in the experiments discussed in detail in Section 4. Of course, we used the Euclidean metric without discretizing the attribute values (the frequency of words). We also tried all normalization methods for the Euclidean metric reported in [14], but none of them improved the classification accuracy.

Although the Euclidean distance measure performed poorly for text classification, we believed that there must be a way to exploit the attributes' numeric nature and the ordinal relationship between their values. This motivated us to incorporate the difference between the frequency of words, which are the basis of the Euclidean distance, in the VDM and ISCDM. Our hypothesis is that the VDM and ISCDM can be improved if they were modified in ways that exploit the difference between the two attribute values that represent the frequency of words in two documents *x* and *y*. Thus, if the difference between the two values is large, the distance should be large, but if the difference is small, the distance should be small too.

Exploiting the difference can be easily done by rewriting the VDM and ISCDM to incorporate (*x*_*a*_ − *y*_*a*_). We call the modified functions Euclidean VDM (EVDM) and Euclidean ISCDM (EISCDM). EVDM is defined as

(12) $$\begin{array}{c} \text{evdm}\left(x_{a},y_{a}\right)={\left(x_{a}-y_{a}\right)}^{\alpha }.\text{vdm}{\left(x_{a},y_{a}\right)}^{\beta }, \end{array}$$

where *α* and *β* are constants used to control the influence of each term on the distance.

We use it to calculate the distance, *d*, between two documents *x* and *y* as follows:

(13) $$\begin{array}{c} d\left(x,y\right)=\sqrt{\sum_{a=1}^{m}\text{evdm}{\left(x_{a},y_{a}\right)}^{2}}. \end{array}$$

Similarly, EISCDM is defined as

(14) $$\begin{array}{c} \text{eiscdm}\left(x_{a},y_{a}\right)={\left(x_{a}-y_{a}\right)}^{\alpha }.\text{iscdm}{\left(x_{a},y_{a}\right)}^{\beta }. \end{array}$$

The distance between two documents is calculated using EISCDM as follows:

(15) $$\begin{array}{c} d\left(x,y\right)=\sqrt{\sum_{a=1}^{m}\text{eiscdm}{\left(x_{a},y_{a}\right)}^{2}}. \end{array}$$

### 3.2. Frequency-Based ISCDM (FISCDM)

In this section, we propose a frequency-based ISCDM (FISCDM). The MNB algorithm (Section 2.2) inspired us to design the FISCDM for text classification, where the probability of the word given the class is multiplied by the frequency of the word (see (9)). Thus, the frequency of the word can serve as a weight of the conditional probability of the word given the class. However, the distance should be inversely proportional to the frequency of terms in the query instance; i.e., if the frequency of the term is large, the distance should be small, but if it is small, the distance should be large. The ISCDM can be easily modified to incorporate this idea. We call the modified ISCDM the frequency-based ISCDM (FISCDM) and it is defined as

(16) $$\begin{array}{c} \text{fiscdm}\left(x_{a},y_{a}\right)={\left(\frac{1}{\left(x_{a}+1\right)}\right)}^{\alpha }\text{iscdm}{\left(x_{a},y_{a}\right)}^{\beta }, \end{array}$$

where *x*_*a*_ is the value of attribute *a* in the query instance and again *α* and *β* are constants used to control the influence of each term on the distance. This distance between two documents *x* and *y* can be defined as

(17) $$\begin{array}{c} d\left(x,y\right)=\sqrt{\sum_{a=1}^{m}\text{fiscdm}{\left(x_{a},y_{a}\right)}^{2}}. \end{array}$$

Although modifying ISCDM in this ways gives substantially better results, as we will see in the next section, doing a similar modification to the VDM (incorporating 1/(*x*_*a*_ + 1)) did not improve its classification accuracy. This modification probably works for ISCDM but not for VDM because ISCDM is a function of the attribute value of the query instance and the class value of the training instance, while VDM is a function of the attribute values of the query and training instances, and all classes.

### 3.3. Combining Both Improvements

If each of the above modifications improves the ISCDM, then combining both may even give better results. Therefore, we combined both ideas in a distance function we call the combined ISCDM (CISCDM) and is defined as follows:

(18) $$\begin{array}{c} d\left(x,y\right)=\sqrt{\sum_{a=1}^{m}\text{ciscdm}{\left(x_{a},y_{a}\right)}^{2}}, \end{array}$$

(19) $$\begin{array}{c} \text{ciscdm}\left(x_{a},y_{a}\right)={\left(\frac{1}{\left(x_{a}+1\right)}\right)}^{\alpha }{\left(x_{a}-y_{a}\right)}^{\beta }.\text{iscdm}{\left(x_{a},y_{a}\right)}^{\gamma }. \end{array}$$

Similar to *α* and *β*, *γ* is a constant used to control the influence the constituent terms iscdm(*x*_*a*_, *y*_*a*_) on the overall distance.

## 4. Experimental Results

We performed several experiments to evaluate the proposed distance functions' effectiveness when used with the kNN algorithm, with *k* = 3 in all experiments. We used 18 benchmark text classification datasets obtained from the Weka [25] website. Table 1 provides a brief description of each dataset in terms of the number of attributes, number of instances, and number of classes (categories). All attributes were discretized using Fayyad and Irani's [26] supervised discretization method as implemented in Weka [25]. All of our algorithms were implemented within the Weka framework. Ten-fold cross-validation was used in all experiments. A paired t-test with a confidence level of 95% was used to determine if each difference was statistically significant. We also used the Wilcoxon signed-rank test to compare the distance functions on all datasets because it is more suitable than the t-test for comparing classifiers over multiple datasets [27].

We used Laplace accuracy terms to approximate the values of *P*(*c*/*y*_*a*_), which is used by the VDM, and *p*(*x*_*a*_/*y*_class_), which is used by the ISCDM. This is done by simply adding 1 to the numerator and the number of values to the denominator of the formulas for *P*(*c*/*y*_*a*_) and *p*(*x*_*a*_/*y*_class_).

### 4.1. Comparing the Proposed Distance Metrics

We evaluated the performance of the VDM and ISCDM on the 18 text classification datasets.  Table 2 summarizes the results. The last two rows show the number of datasets for which the measures achieved better results and the number of signiﬁcantly better results at a 95% conﬁdence level. The average classification accuracies of the VDM and ISCDM are 79.72% and 86.30%, respectively. Furthermore, the ISCDM outperforms the VDM for 15 datasets, achieving significantly better results for 11 of these datasets. On the other hand, the VDM produces better results for three datasets, only two of which are significantly better results. We also performed the Wilcoxon signed-rank test, which also showed that the results on all datasets were statistically significant at a 99% confidence level.

Table 2 also shows a comparison between the VDM and EVDM with *α* and *β* set to 1 and 3, respectively. These values were determined empirically. The results reveal that the EVDM has an average classification accuracy of 83.04%, which is higher by 3.32% than the VDM's average accuracy. The EVDM achieves better results than the VDM for 14 datasets, 12 of which are statistically significant results, while the VDM achieves better results for four datasets, only two of which are statistically significant results. Moreover, the Wilcoxon signed-rank test shows that these results are statistically significant at 99% confidence level.

Comparing EVDM and ISCDM shows, however, that ISCDM is still the superior distance function. It has higher average accuracy than the EVDM by 3.27%. It also achieves better results than EVDM for 13 datasets, 10 of which are statistically significant results. EVDM, on the other hand, produces better results for five datasets, three of which are significantly better results. The Wilcoxon signed-rank test shows that these results are statistically significant at 95% confidence level.

**Figure 1:**
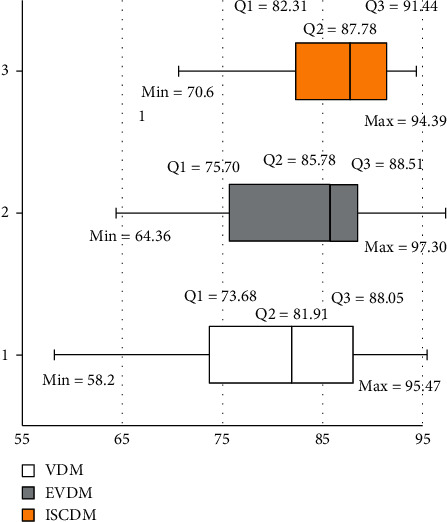
Boxplot for VDM, EVDM, and ISCDM.

Figure 1 shows the boxplots for the VDM, EVDM, and ISCDM. It shows that, compared to VDM, EVDM has a higher minimum, first quartile, second quartile (median), third quartile, and maximum value. For example, VDM's median accuracy is 81.91%, while the median of the EVDM is 85.78%. This, of course, means that while 50% of the results of the VDM are higher than 81.91%, 50% of the results of the EVDM are higher than 85.78%.

Figure 1 also shows that the ISCDM has a higher minimum, first quartile, second quartile, and third quartile values than the EVDM. However, the EVDM has higher maximum values than the ISCDM. Comparing the first quartile values of the ISCDM and EVDM shows that 75% of the classification accuracies of the ISCDM and the EVDM for all datasets are higher than 82.31% and 75.70%, respectively. Comparing the medians reveals that 50% of the results of the ISCDM and EVDM are higher than 87.78% and 85.78%, respectively, while comparing the third quartile values shows that 25% of the results of the ISCDM and the EVDM have higher classification accuracy than 91.44% and 88.51%, respectively.

We also compared the ISCDM with its modified versions, namely, the EISCDM, FISCDM, and CISCDM. The EISCDM was used with *α* and *β* values of 1 and 0.5, respectively, while the FISCDM was used with *α* and *β* values of 0.5 and 1, respectively. The CISCDM gave the best results with *α*, *β*, and *γ* of 0.5, 1, and 1, respectively. All these values were determined empirically.

Table 3 shows the results of each of the proposed functions compared to the result of the ISCDM. The results reveal that each improved function achieves significantly better results than the ISCDM for most datasets. The EISCDM achieves better results than the ISCDM for 13 datasets, of which 11 are significantly better results, while the ISCDM achieves better results than EISCDM for five datasets, of which only two are significantly better results. The FISCDM, on the other hand, achieves better results than the ISCDM for 13 datasets, 10 of which are significantly better results, while the ISCDM achieves better results for four datasets, but none of them is significantly a better result. The best improvement, however, comes from the CISCDM. It achieves better results for 13 datasets, 11 of which are significantly better results, while the ISCDM achieves better results for only two datasets, but none of these is a significantly better result.

**Figure 2:**
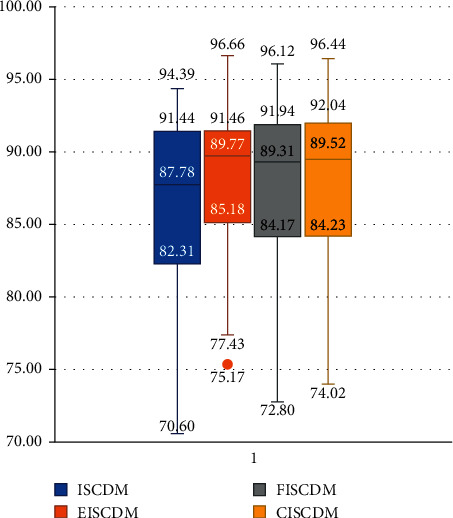
Comparing ISCDM, EISCDM, FISCDM, and CISCDM.

Figure 2 shows a boxplot for the ISCDM, EISCDM, FISCDM, and CISCDM. The boxplot shows that all the proposed measures also have a higher minimum, first quartile, second quartile, third quartile, and maximum results (classification accuracies) than the ISCDM. The boxplot also shows that the EISCDM has a higher minimum, first quartile, second quartile, and maximum results than both the FISCDM and CISCDM. However, it has a lower third quartile than both of them.

**Figure 3:**
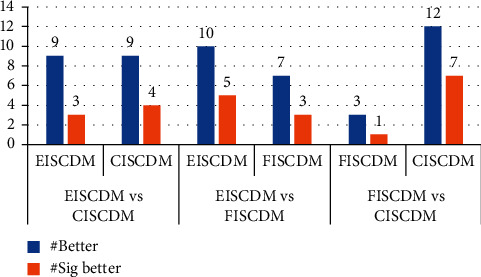
Comparing the EISCDM, FISCDM, and CISCDM.

To determine the best measure, we compared the EISCDM, FISCDM, and CISCDM directly with each other in terms of the number of datasets for which they achieve better and significantly better results.  Figure 3 summarizes our comparison results. It shows that the CISCDM and EISCDM achieve better results for more datasets than the FISCDM. However, the CISCDM outperforms the FISCDM in more obvious ways than EISCDM does.  Figure 3 also shows that the CISCDM achieves significantly better results than the EISCDM for four datasets, while the EISCDM achieves significantly better results than CISCDM for three datasets, which is a close result. Moreover, as Table 3 shows, the CISCDM also outperforms the ISCDM in more obvious ways than EISCDM. The table shows that CISCDM and EISCDM achieve significantly better results than the ISCDM for 11 datasets; however, while the ISCDM does not achieve significantly better results than the CISCDM for any dataset, it achieves significantly better results than the EISCDM for two datasets. For all these reasons, we consider the CISCDM to be better than EISCDM. However, it is worth mentioning that, unlike FISCDM and CISCDM, both EVDM and EISCDM can be used with any discretized numeric attributes and not just term-frequencies. They both only require the existence of an ordinal relationship between values, which is preserved when a numeric attribute is discretized.

In Section 4.2, we compare the CISCDM with the Naïve Bayesian-based text classifiers.

It is also worth mentioning that the improved methods are not very sensitive to the values of the constants *α*, *β*, and *γ*.

Table 4 shows the results we obtained when we set each one of them to 1. Each entry in the table shows the result in wins/ties/losses notation. We consider a dataset a win only if the improved distance function achieves a significantly better result for that dataset at a 95% confidence level. The table shows that each proposed distance function achieves better average accuracy and better accuracy for more datasets than its unmodified counterpart.

### 4.2. Comparing CISCDM with Naïve Bayesian-Based Text Classification Algorithms

**Figure 4:**
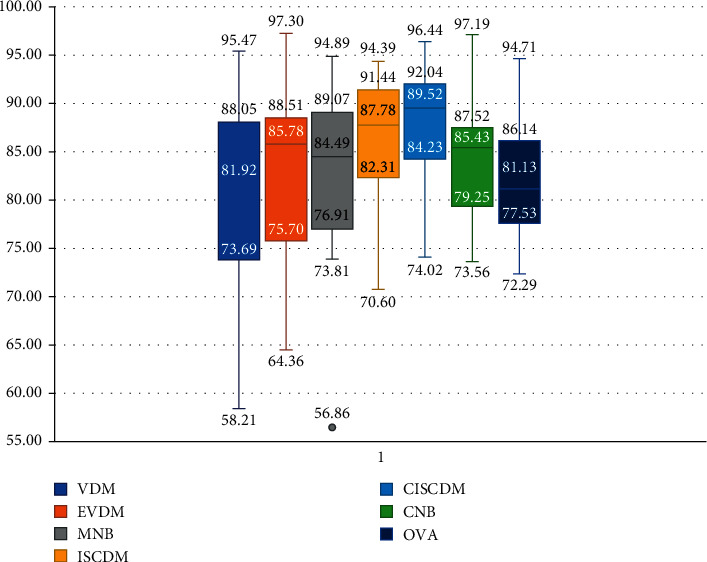
Boxplot of the proposed distance measures and NB-based text classifiers.

This section compares the CISCDM with the Bayesian-based methods described in Section 2.2, namely, MNB, CNB, and OVA. According to [24], some of these heuristic methods' accuracy approaches, the accuracy of state-of-the-art text classification methods, such as support vector machines.  Table 5 shows the results of comparing the ISCDM and CISDM with MNB, CNB, and OVA. The results show that while the ISCDM performs well compared to all NB-based text classifiers, the CISCDM performs even better. The CISCDM has better average classification accuracy and gives significantly better accuracy for more datasets compared to all other classifiers. The CISCDM also has better first, second, and third quartiles (see Figure 4) than all other text classifiers. The ISCDM is in second place with better first, second, and third quartiles than all NB-based text classifiers.

The main drawback of the kNN and lazy machine learning methods, in general, is their long classification time. However, these drawbacks can be mitigated using instance reduction techniques [23, 28] or instance, indexing techniques [29].

## 5. Conclusion

In this paper, we proposed new distance measures for text classification that are based on VDM and ISCDM. The new distance measures exploit word frequencies and/or the ordinal relationship between them. We evaluated the measures using the kNN algorithm (with *k* = 3) on 18 benchmark text classification datasets. Our results indicate that all improved distance functions achieve significantly better results for most datasets than their unmodified counterparts. We also compared ISCDM and CISCDM with Naïve Bayesian-Based text classifiers, namely, MNB, CNB, and OVA. Our empirical results show that although they both achieve better results than the Bayesian methods, the proposed CISCDM is the superior distance measure. In fact, some of the proposed distance measures can work for domains other than text classification, namely, EVDM and EISCDM. They work for any discretized numeric attributes because all they assume is an ordinal relationship between the discretized values. Evaluating the performance of the EVDM and EISCDM in other domains is a subject for future research. Future work may investigate using instance reduction and indexing techniques to speed up the classification process. It may also be interesting to use evolutionary methods for determining the best values for the constants *α*, *β*, and *γ* and for determining better estimations of the probability terms used by the distance measures [30] and NB-based text classifiers [31, 32].

## Figures and Tables

**Table 1:** A description of the benchmark text classification datasets used in the empirical work.

Datasets	#Atts	#Insts	#Classes
Fbis	2001	463	17
la1s	13196	3204	6
la2s	12433	3075	6
Oh0	3183	1003	10
Oh10	3239	1050	10
Oh15	3101	918	10
Oh5	3013	918	10
Ohscal	11466	11,163	10
Re0	2889	1504	13
Re1	3759	1658	25
Tr11	6430	414	9
Tr12	5805	313	8
Tr21	7903	336	6
Tr23	5833	204	6
Tr31	10129	927	7
Tr41	7455	878	10
Tr45	8262	690	10
Wap	8461	1561	20

**Table 2:** Pairwise comparison between VDM, ISCDM, and EVDM.

Dataset	VDM vs. ISCDM		VDM vs. EVDM		EVDM vs. ISDCM	
	VDM	ISCDM	VDM	EVDM	EVDM	ISCDM
fbis.wc	**74.75**	70.60	74.75	**76.61**	**76.61**	70.60
la1s.wc	65.04	**87.05**	65.04	**73.94**	73.94	**87.05**
la2s.wc	66.05	**87.54**	66.05	**73.82**	73.82	**87.54**
Oh0.wc	78.27	**91.63**	78.27	**87.64**	87.64	**91.63**
Oh5.wc	83.99	**88.02**	83.99	**87.36**	87.36	**88.02**
Oh10.wc	73.33	**80.95**	73.33	**78.86**	78.86	**80.95**
Oh15.wc	79.85	**85.54**	79.85	**83.35**	83.35	**85.54**
ohscal.wc	63.04	**74.11**	63.04	**73.09**	73.09	**74.11**
re0.wc	79.79	**80.12**	**79.79**	75.40	75.40	**80.12**
re1.wc	**85.88**	84.25	**85.88**	84.97	**84.97**	84.25
tr11.wc	87.68	**88.41**	87.68	**88.65**	**88.65**	88.41
tr12.wc	88.18	**92.01**	**88.18**	86.58	86.58	**92.01**
tr21.wc	89.29	**93.75**	**89.29**	88.10	88.10	**93.75**
tr23.wc	89.71	**92.16**	89.71	**91.18**	91.18	**92.16**
tr31.wc	**95.47**	94.39	95.47	**97.30**	**97.30**	94.39
tr41.wc	90.21	**90.89**	90.21	**94.42**	**94.42**	90.89
tr45.wc	86.38	**90.43**	86.38	**89.13**	89.13	**90.43**
wap.wc	58.21	**81.67**	58.21	**64.36**	64.36	**81.67**
**Average**	**79.73**	**86.31**	**79.72**	**83.04**	**83.04**	**86.31**
**#Better**	3	15	4	14	5	13
**#Sig better**	2	11	2	12	3	10

**Table 3:** Improved specific-class distance measures.

Dataset	EISCDM vs. ISCDM		FISCDM vs. ISCDM		CISDM vs. ISCDM	
	ISCDM	EISCDM	ISCDM	FISCDM	ISCDM	CISCDM
fbis.wc	70.60	**77.43**	70.60	**72.80**	70.60	**74.02**
La1s.wc	87.05	**89.51**	87.05	**88.92**	87.05	**89.33**
La2s.wc	87.54	**90.24**	87.54	**89.69**	87.54	**89.72**
Oh0.wc	**91.63**	90.03	91.63	**92.02**	91.63	91.63
Oh5.wc	88.02	**88.67**	88.02	**88.13**	88.02	88.02
Oh10.wc	**80.95**	80.67	80.95	80.95	80.95	**81.33**
Oh15.wc	**85.54**	85.32	85.54	**85.65**	85.54	**85.76**
ohscal.wc	74.11	**75.17**	74.11	**74.85**	74.11	**76.68**
re0.wc	80.12	**82.31**	**80.12**	79.85	80.12	**81.18**
re1.wc	84.25	**85.15**	84.25	**85.52**	84.25	**85.94**
tr11.wc	88.41	**90.58**	88.41	**89.86**	88.41	**90.10**
tr12.wc	**92.01**	91.05	**92.01**	91.69	**92.01**	91.69
tr21.wc	**93.75**	91.96	**93.75**	93.15	**93.75**	93.15
tr23.wc	92.16	**93.14**	**92.16**	91.67	92.16	92.16
tr31.wc	94.39	**96.66**	94.39	**96.12**	94.39	**96.44**
tr41.wc	90.89	**93.39**	90.89	**93.39**	90.89	**94.08**
tr45.wc	90.43	**91.59**	90.43	**92.75**	90.43	**92.61**
wap.wc	81.67	**85.26**	81.67	**83.72**	81.67	**83.72**
Average	86.31	87.68	86.31	87.27	86.31	87.65
#Better	5	13	4	13	2	13
#Sig Better	2	11	0	10	0	11

**Table 4:** Comparing the distance metrics with their improved versions (with *α*, *β*, and *γ* set to 1).

Distance function	EVDM		EISCDM		FISCDM		CISCDM	
	Average	W/T/L	Average	W/T/L	Average	W/T/L	Average	W/T/L
VDM	80.49	7/10/1	—	—	—	—	—	—
ISCDM	—	—	86.99	11/5/2	87.11	6/9/3	87.41	10/5/3

**Table 5:** Comparing ISCDM and CISCDM with Bayesian-based text classifiers.

Dataset	MNB vs. ISCDM		MNB vs. CISCDM		CNB vs. ISCDM		CNB vs. CISCDM		OVA vs. ISCDM		OVA vs. CISCDM	
	MNB	ISCDM	MNB	CISCDM	CNB	ISCDM	CNB	CISCDM	OVA	ISCDM	OVA	CISCDM
fbis.wc	**73.81**	70.60	73.81	**74.02**	**73.56**	70.60	73.56	**74.02**	**79.17**	70.60	**79.17**	74.02
la1s.wc	**89.08**	87.05	89.08	**89.33**	86.20	**87.05**	86.20	**89.33**	86.39	**87.05**	86.39	**89.33**
la2s.wc	**89.04**	87.54	89.04	**89.72**	**87.61**	87.54	87.61	**89.72**	**87.84**	87.54	87.84	**89.72**
oh0.wc	**92.92**	91.63	**92.92**	91.63	**92.32**	91.63	**92.32**	91.63	90.92	**91.63**	90.92	**91.63**
oh5.wc	**88.78**	88.02	**88.78**	88.02	**88.46**	88.02	**88.46**	88.02	84.97	**88.02**	84.97	**88.02**
oh10.wc	**83.71**	80.95	**83.71**	81.33	**82.00**	80.95	**82.00**	81.33	80.76	**80.95**	80.76	**81.33**
oh15.wc	**86.53**	85.54	**86.53**	85.76	**85.77**	85.54	**85.77**	85.76	81.50	**85.54**	81.50	**85.76**
ohscal.wc	**75.41**	74.11	75.41	**76.68**	**77.55**	74.11	**77.55**	76.68	**76.98**	74.11	**76.98**	76.68
re0.wc	73.87	**80.12**	73.87	**81.18**	77.47	**80.12**	77.47	**81.18**	75.15	**80.12**	75.15	**81.18**
re1.wc	81.41	**84.25**	81.41	**85.94**	78.69	**84.25**	78.69	**85.94**	72.29	**84.25**	72.29	**85.94**
tr11.wc	84.06	**88.41**	84.06	**90.10**	84.52	**88.41**	84.52	**90.10**	80.17	**88.41**	80.17	**90.10**
tr12.wc	**94.89**	92.01	**94.89**	91.69	86.92	**92.01**	86.92	**91.69**	76.67	**92.01**	76.67	**91.69**
tr21.wc	74.40	**93.75**	74.40	**93.15**	85.09	**93.75**	85.09	**93.15**	80.03	**93.75**	80.03	**93.15**
tr23.wc	56.86	**92.16**	56.86	**92.16**	80.95	**92.16**	80.95	**92.16**	85.38	**92.16**	85.38	**92.16**
tr31.wc	92.77	**94.39**	92.77	**96.44**	**97.19**	94.39	**97.19**	96.44	**94.71**	94.39	94.71	**96.44**
tr41.wc	**91.00**	90.89	91.00	**94.08**	**92.15**	90.89	92.15	**94.08**	89.30	**90.89**	89.30	**94.08**
tr45.wc	84.93	**90.43**	84.93	**92.61**	87.25	**90.43**	87.25	**92.61**	82.17	**90.43**	82.17	**92.61**
wap.wc	**82.76**	81.67	82.76	**83.72**	76.60	**81.67**	76.60	**83.72**	73.91	**81.67**	73.91	**83.72**
Average	83.13	86.31	83.13	87.65	84.46	86.31	84.46	87.65	82.13	86.31	82.13	87.65
#Better	**11**	**7**	**5**	**13**	**9**	**9**	**6**	**12**	**4**	**14**	**2**	**16**
#Sig Better	**6**	**6**	**1**	**9**	**3**	**8**	**1**	**10**	**2**	**10**	**1**	**13**

## Data Availability

All datasets used are publically available and the sources are properly cited in the paper.
